# Heterosis and Responses to Selection in Orange-Fleshed Sweetpotato (*Ipomoea batatas L.*) Improved Using Reciprocal Recurrent Selection

**DOI:** 10.3389/fpls.2022.793904

**Published:** 2022-04-26

**Authors:** Wolfgang J. Grüneberg, Bert De Boeck, Federico Diaz, Raul Eyzaguirre, Jan W. Low, Jochen C. Reif, Hugo Campos

**Affiliations:** ^1^International Potato Center (CIP), Lima, Peru; ^2^CIP-Sub-Saharan Africa, Nairobi, Kenya; ^3^Leibniz Institute of Plant Genetics and Crop Plant Research (IPK), Gatersleben, Germany

**Keywords:** heterosis, response to selection, reciprocal recurrent selection, population hybrid breeding, orange-fleshed sweetpotato

## Abstract

Sweetpotato is a highly heterozygous hybrid, and populations of orange-fleshed sweetpotato (OFSP) have a considerable importance for food security and health. The objectives were to estimate heterosis increments and response to selection in three OFSP hybrid populations (H_1_) developed in Peru for different product profiles after one reciprocal recurrent selection cycle, namely, H_1_ for wide adaptation and earliness (O-WAE), H_1_ for no sweetness after cooking (O-NSSP), and H_1_ for high iron (O-HIFE). The H_1_ populations were evaluated at two contrasting locations together with parents, foundation (parents in H_0_), and two widely adapted checks. Additionally, O-WAE was tested under two environmental conditions of 90-day and a normal 120-day harvest. In each H_1_, the yield and selected quality traits were recorded. The data were analyzed using linear mixed models. The storage root yield traits exhibited population average heterosis increments of up to 43.5%. The quality traits examined have exhibited no heterosis increments that are worth exploiting. The storage root yield genetic gain relative to the foundation was remarkable: 118.8% for H_1_-O-WAE for early harvest time, 81.5% for H_1_-O-WAE for normal harvest time, 132.4% for H_1_-O-NSSP, and 97.1% for H_1_-O-HIFE. Population hybrid breeding is a tool to achieve large genetic gains in sweetpotato yield *via* more efficient population improvement and allows a rapid dissemination of globally true seed that is generated from reproducible elite crosses, thus, avoiding costly and time-consuming virus cleaning of elite clones typically transferred as vegetative plantlets. The population hybrid breeding approach is probably applicable to other clonally propagated crops, where potential for true seed production exists.

## Introduction

Sweetpotato [*Ipomoea batatas* (L.) Lam.] is widely grown in the tropics and subtropics on approximately 8 million ha, with a storage root yield average of 11.9 t ha^–1^ (FAOSTAT, 2018). It has a short crop duration (120–150 days), large flexibility in harvest times, and a pronounced tolerance to biotic and, especially, abiotic stresses (Grüneberg et al., 2015). The orange-fleshed sweetpotato (OFSP) has very high root β-carotene contents, which the body converts into bioavailable vitamin A (van Jaarsveld et al., 2005; Bovell-Benjamin, 2007; Pfeiffer and McClafferty, 2007). The OFSP was the first biofortified staple for vitamin A to be delivered at large scale (Bouis and Saltzman, 2017) in sub-Saharan Africa, and the current breeding efforts aim at increasing its iron content, with the goal of developing stacked vitamin A, iron, plus zinc biofortified OFSP (Low et al., 2020).

The storage root yield is used mainly for home consumption, retail fresh markets, and in the food processing industry; the latter use is widespread, so far, only in China (Woolfe, 1992; Bovell-Benjamin, 2007; Padmaja, 2009). A part of the aboveground biomass is needed as a planting material because sweetpotato is propagated by vine cuttings. The remaining foliage is often used as an animal feed – fresh, dried, or as silage – in many countries, leaves are also consumed as a green vegetable. The major product profiles for the OFSP market sector are (i) OFSP for wide adaptation and earliness (O-WAE) with very short crop duration; (ii) OFSP with high dry matter content and a strong resistance to sweetpotato virus disease (SPVD) for East Africa; (iii) non-sweet sweetpotato (O-NSSP) with no or low sweet taste after cooking, demanded by small-scale and industrial food processing and some consumer segments; and (iv) an OFSP double biofortified with high iron (O-HIFE) to address the most common major micronutrient deficiency worldwide. The OFSP product profiles described in this study are currently targeting about 759,000 hectares in six OneCGIAR regions and 30 countries, respectively (Sylvester Ojwang personal communication, 2021)– note: O-NSSP and O-HIFE are new product profiles to expand the OFSP market sector. The major challenges in breeding are to boost genetic gains, as in other clonal crops, and extreme delays in getting virus-free products out of the country of origin due to phytosanitary issues.

Sweetpotato is an autopolyploid (6 x = 90, x = 15), a highly heterozygous clone hybrid, with an easy true seed generation set by out-crossing (a successful pollination results in 1–3 true seeds). The plant has a sporophytic self-incompatibility system (Martin and Cabanillas, 1966; Martin, 1968). It is hypothesized that heterosis greatly contributes to the performance of sweetpotato (Gallais, 2003). Mid-parent heterosis can be assessed using either populations or heterozygous or homozygous clones as parents. Studies about mid-parent heterosis are very limited in sweetpotato and were estimated exclusively using heterozygous clones as parents. Grüneberg et al. (2015) reported a mid-parent heterosis ranging from −34 to 58% for storage root yield of 48 F_1_ hybrids derived from factorial crosses of 4 × 12 clones. Kivuva et al. (2015) observed a range of mid-parent heterosis for storage root yield from −43 to 92% under no-drought stress and −54 to 82% under drought stress in 15 F_1_ hybrids derived from an applied breeding population. Diaz et al. (2021) reported mid-parent heterosis for storage root yields ranging from −30.6 to 139.4%, with a mean of 21.8% in 210 F_1_ hybrids, tracing back to two parental gene pools. The extent of heterosis is, thus, considerable, and its systematic exploitation deserves a deeper consideration as a novel avenue to increase the productivity and climate resilience.

Heterosis can be systematically exploited, applying reciprocal recurrent selection (RRS), a cyclic breeding procedure, in which the hybrid performance from crosses between two parental populations is improved by the selection of the best combining genotypes in each population to generate new parents in each parental population for the next breeding cycle (Hallauer, 1992). The term was first coined by Hull (1945), who focused on maize but was already proposing the use of RRS in clonal crops, using the complex polyploid sugar cane as an example. So far, RRS has received little attention in clonal crop breeding (Melchinger and Gumber, 1998), with first attempts in sweetpotato (Grüneberg et al., 2009). Recently, RRS has been more intensively discussed for the foliage crops (Miles, 2007), potato (Lindhout et al., 2011; Jansky et al., 2016), and sweetpotato (David et al., 2018). In sweetpotato, RRS has been implemented at the International Potato Center (CIP) in Peru since 2010, applying a population hybrid breeding approach (David et al., 2018), and aims to breed superior heterozygous clones as final varieties. Unique plant material was developed, comprising three sweetpotato hybrid populations that were improved in an RRS cycle. The hybrids were evaluated with their parents and founder clones to test the hypothesis that the response to selection is larger in interpopulation compared to the more widely used intrapopulation improvement approach, with an estimated annual genetic gain for the latter of 0.8–2.5% for storage root yield across the four breeding platforms over the past decade (CIP International Potato Center, 2020). Two widely grown modern varieties (Dagga and Cemsa_74-228) were used to determine the variety ability (Gallais, 2003) of the hybrid populations by the frequency of hybrid offspring clones, surpassing these two mega-clones. The objectives were to (i) examine the extent of heterosis in population crosses, (ii) to implement and to evaluate the response to selection of RRS in three OFSP hybrid populations (H_1_-O-WAE, H_1_-O-NSSP, and H_1_-O-HIFE), and (iii) to obtain information on the variety ability of the three advanced hybrid populations.

## Materials and Methods

### Reciprocal Recurrent Selection Program

**FIGURE 1:**
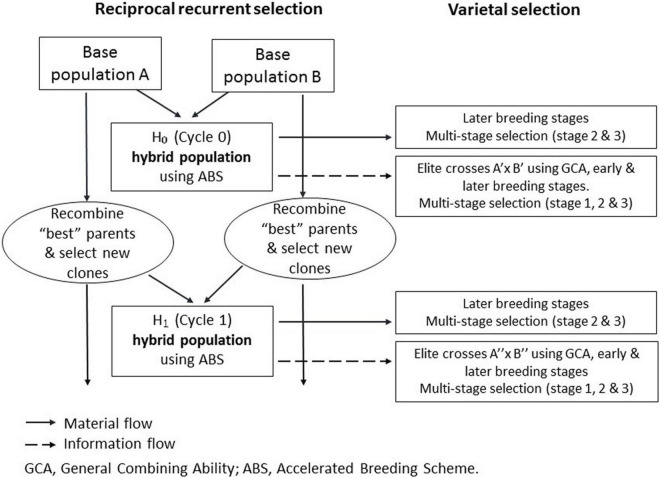
The International Potato Center (CIP)’s approach to population hybrid breeding with reciprocal recurrent selection **(left)** and hybrid variety selection **(right)** – a comprehensive breeding scheme as outlined by Gallais (2003), with selection of best parents on the basis of their general combining ability effects.

The RRS program traces back to the two OFSP breeding populations developed in Peru and designated as PJ and PZ, which were both established to breed for storage root yield, wide adaptation, elevated root β-carotene, and medium-to-high root dry matter contents. The PJ and PZ pools are clearly distinguishable using pedigree and molecular marker data. Together, they cover nearly the entire diversity of sweetpotato, anchored by 21 mega-clones, and are mutually heterotic (Diaz et al., 2021). To establish the RRS program (Figure 1), 49 PJ and 31 PZ founder clones were selected (Supplementary Table 1). From these clones, a PJ × PZ hybrid population 0 (H_0_) was developed in Cycle 0 (C_0_), and H_0_ hybrids (see Figure 1, top left) were evaluated at two sites: (i) Huaral (11°31′S, 77°14′W) on the arid Pacific coast, corresponding to a Mediterranean temperate climate; and (ii) San Ramon (11°07′S, 75°21′W) in the humid tropics of the Amazon basin [for details concerning sites and trials, see Diaz et al. (2021)].

Using the H_0_ offspring information and according to the three product profiles, i.e., O-WAE, O-NSSP, and O-HIFE, six different sets of clones were selected for intra-gene pool crossings in Cycle 1 (C_1_). Selection criteria among founder clones are as follows: (i) for O-WAE, across H_0_ half-sibs, storage root yield larger than H_0_ population mean and mean heterosis for storage root yield larger than zero; (ii) for O-NSSP, across H_0_ half-sibs, low root sucrose, mean heterosis for storage root yield larger than zero, and storage root yield larger than or close to H_0_ population mean; and (iii) for O-HIFE, across H_0_ half-sibs, high root iron, mean heterosis for storage root yield larger than zero, and storage root yield larger than or close to H_0_ population mean. Applying these criteria, 23 PJ and 17 PZ clones were selected for O-WAE, five PJ and five PZ for O-NSSP, and five PJ and five PZ for O-HIFE (Supplementary Table 1).

The recombination of intra-gene pool parents resulted in the generation of 9,324 PJ′ and 2,152 PZ′ clones for O-WAE, 702 PJ′ and 379 PZ′ clones for O-NSSP, and 1,006 PJ′ and 711 PZ′ clones for O-HIFE. The intra-gene pool progenies were evaluated in the field trials at three locations in Peru (San Ramon, previously described, and two sites on the arid Pacific coast: (i) Motupe, 6°9′S, 79°42′W, and (ii) Cañete, 13°04′S, 76°23′W). The following traits were assessed: (i) storage root yield, agronomic score value of storage roots, and dry matter content for O-WAE; (ii) storage root yield, agronomic score value of storage roots, dry matter content, root starch, root sucrose, β-carotene content, and root iron for O-NSSP; and (iii) storage root yield, agronomic score value of storage roots, dry matter content, root starch, root sucrose, β-carotene content, and root iron for O-HIFE. Quality traits were determined using a near-infrared spectrometry (NIRS), after freeze-drying the raw storage root samples following the procedures described by Tumwegamire et al. (2011). The traits were combined using the index of desired gains by Pesek and Baker (1969) in a modification (see Supplementary Information for more details). Based on the index and considering the vine vigor, SPVD symptoms, and hybrid production traits (true seed set), 41 PJ′ and 41 PZ′ (O-WAE), 25 PJ′ and 28 PZ′ (O-NSSP), and 28 PJ′ and 28 PZ′ (O-HIFE) clones were selected (PJ′ and PZ′ parents of the first selection cycle) to establish the three H_1_ hybrid populations: H_1_-O-WAE, H_1_-O-NSSP, and H_1_-O-HIFE. Note that the selection intensity at this step was very high with α of 0.0044 and 0.0191 for O-WAE in PJ′ and PZ′, respectively, and, correspondingly, 0.0356 and 0.0739 for O-NSSP, and 0.0278 and 0.0394 for O-HIFE.

### Evaluation of Selection Gain in Field Trials

The selected PJ′ and PZ′ clones were crossed, and three H_1_ populations were developed that comprised 9,881 H_1_-O-WAE clones, representing 742 out of the 1,681 possible single-crosses between the parental clones (in case of no incompatibility and true seed set capabilities), 3,742 H_1_-O-NSSP clones representing 336 out of the 700 possible single-crosses between the parental clones, and 3,292 H_1_-O-HIFE clones representing 272 out of the 784 F_1_ possible single-crosses between the parental clones. The hybrid populations were evaluated together with their parents, PJ′ and PZ′, and the founder clones PJ and PZ in three neighboring experiments at each location. The trials at Canete for H_1_-O-NSSP and H_1_-O-HIFE were directly in neighbor, whereas the trial for H_1_-O-WAE 90- and 120-day harvest at Canete was located at about 200 m distance (this trial for H_1_-O-WAE 90 and 120 days was already about 5 hectares). At Satipo, all three experiments (H_1_-O-WAE for 90- and 120-day harvest, H_1_-O-NSSP and H_1_-O-HIFE) were directly in neighbor. The environments are contrasting: (i) Cañete (previously described) and (ii) Satipo in the humid tropics of the Amazon basin (11°15′S; longitude, 74°38′W). The 49 PJ and 31 PZ founder clones were identical for each H_1_ population. Two modern and widely adapted varieties were used as checks in the field trials: light orange-fleshed Dagga (CIP199062.1) from Peru and pale yellow-fleshed Cemsa_74-228 (CIP400004) from Cuba. The checks and founder clones are available from CIP’s gene bank, and parental clones for each H_1_ offspring are in the process of becoming available.

The field trials consisted of 1-m long row plots with three planting positions per plot, with an exception for H_1_-O-WAE trials, which were planted as double plots (2 × 1-m rows) to harvest half of the double plot at 90 days after planting, and the second half at 120 days. For all parents and all founder clones, eight 1-m plot replications were used, whereas H_1_ offspring clones were planted without plot replications. All 1-m test plots for offspring, parents, and foundation were completely randomized in each field trial, whereas the 1-m plots for the two check clones were planted in a grid using Dagga and Cemsa_74-228 alternated within the grid rows and alternated to the left and right sides of 10 test plot rows (total size of experiments was approximately 3.5, 0.75, and 0.7 hectares for the H_1_-O-WAE, H_1_-O-NSSP, and H_1_-O-HIFE trials, respectively, at each location). This principle of the design was described by Westcott (1981). In Cañete and Satipo, H_1_-O-WAE field trials were planted on June 23 and August 2, 2017, respectively, and were harvested in 90 and 120 days after planting, during the winter season at the Peruvian coast. Correspondingly, H_1_-O-NSSP trials were planted on April 21 and August 11, 2017, and H_1_-O-HIFE trials were planted on February 2 and June 2, 2017, and both types were harvested in 120 days after planting.

The traits recorded in H_1_-O-WAE field trials are as follows: storage root yield (t ha^–1^), number of commercial storage roots per plant by dividing the number of commercial storage roots per plot by the number of harvested plants per plot, foliage yield (t ha^–1^), dry matter content (%) from root dry weight divided by root fresh weight, and β-carotene contents [on an mg 100-g^–1^ root dry weight basis (dwb)] – color charts were used to first determine the β-carotene content on a root fresh weight basis (fwb) as described by Burgos et al. (2009). Each trait was recorded at 90 and 120 days after harvest and recorded as repeated measurements. The traits recorded in the H_1_-O-NSSP trials are as follows: storage root yield (t ha^–1^), number of commercial storage roots per plant, foliage yield (t ha^–1^), dry matter content (%), β-carotene contents (mg 100 g^–1^dwb) using color charts, and sweetness taste after cooking (COSW) on a 1–9 scale (1 is the lowest sweetness score). The traits recorded in H_1_-O-HIFE trials are as follows: storage root yield (t ha^–1^), number of commercial storage roots per plant, foliage yield (t ha^–1^), dry matter content (%), and β-carotene contents (mg 100 g^–1^dwb) using NIRS, root starch (%), and iron (mg kg^–1^). The quality traits in the H_1_-O-HIFE trials were determined by NIRS as described by Tumwegamire et al. (2011). Data are available as open access from SweetPotatoBase (2021) https://sweetpotatobase.org/folder/4366.

### Data Analysis

Statistical analysis was carried out as a two-stage analysis using linear mixed models to analyze the phenotypic data obtained for each H_1_ population, together with its parental material, founders, and checks. Such a stage-wise approach to phenotypic multi-environment trial data analyses was extensively described by van Eeuwijk et al. (2016) and Piepho et al. (2012). Separate but identical analyses were performed for the different traits and populations using an R software (R Core Team, 2019). To simplify the notation, the methodology is elaborated for a single trait and a single population (without indexing).

In the first stage, the adjusted best linear unbiased estimations (BLUEs) for all genotypes were determined for both environments (*j* = 1, 2) separately using the following spatial mixed model (for the *j*th environment):


$$y_{i\,k\,l\,\left(j\right)}=\mu_{i\,\left(j\right)}+r_{k\,\left(j\right)}+c_{l\,\left(j\right)}+f_{j}\,\left({\text{x}}_{k},{\text{x}}_{l}\right)+\varepsilon_{i\,k\,l\,\left(j\right)},$$

where *y*_*ikl(j)*_ (*j* = 1,2;*i* = 1,..,*p*;*k* = 1,..,*n*_*r*_;*l* = 1,..,*n*_*c*_) is the phenotypic response of the *i*th genotype in the *k*th row and *l*th column, μ_*i*(*j*)_ the*i*th (fixed) genotype mean, $r_{k\,\left(j\right)}\,∼\,N\,\left(0,\sigma_{r\,\left(j\right)}^{2}\right)\,$ the random row effect, $c_{l\,\left(j\right)}∼\,N\,\left(0,\sigma_{c\,\left(j\right)}^{2}\right)\,$ the random column effect, *f*_*j*_(x*_k_*,x*_l_*) a smooth bivariate surface defined over the *n*_*r*_ row and *n*_*c*_ column positions of the plots, $\varepsilon_{i\,k\,l\,\left(j\right)}\,∼\,N\,\left(0,\sigma_{\left(j\right)}^{2}\right)\,$ the random error term, and this all in the *j*th environment. The row and column coordinates x*_k_* and x*_l_* are the centered and scaled equivalent of the indices of the *k*th row and *l*th column, respectively. The smooth two-dimensional surface can be further decomposed in a fixed (linear) and random (smooth) part as described by Velazco et al. (2017). This spatial modeling using two-dimensional penalized spline (P-spline) ANOVA mixed models was performed using the R package, “SpATS” (Rodriguez-Alvarez et al., 2018), and was first introduced by Lee et al. (2013).

Averaging out the BLUEs of the hybrids on a family basis (leading to the mid-offspring value per family) and calculating the mid-parent value as the mean of the two parental BLUEs for each family permits determining the mid-parent–mid-offspring Pearson correlations across families for each population, trait, and trial. Averaging out the female parental BLUEs, the male parental BLUEs, and the mid-offspring values (the latter across families) reveals the first view of heterosis increments per population, trait, and trial, respectively.

In the second stage, the genotype-by-environment table of BLUEs (i.e., genotype means or adjusted phenotypes per environment) and their standard errors were used as a starting point for the multi-environment trial analysis. The BLUEs were weighted according to Method 2 of Möhring and Piepho (2009) to fit the following mixed model that considers a possible genotype-by-environment interaction:


$${\hat{\mu }}_{i\,\left(j\right)}=\mu +e_{j}+g_{t}+g\,e_{j\,t}+G_{j\,i}+\varepsilon_{i\,j},$$

where ${\hat{\mu }}_{i\,\left(j\right)}$ (*j* = 1,2;*i* = 1,..,*p*) is the reweighted BLUE of the *i*th genotype (belonging to the *t*th group; not explicitily denoted) in the *j*th environment, *e*_*j*_ the*j*th environment (fixed) main effect, *g*_*t*_ the fixed group effect with levels *t* = 1,..,7 (representing fixed effects for checks Cemsa_74-228 and Dagga, foundation groups PJ and PZ, parental groups PJ′ and PZ′, and hybrids H_1_), *ge*_*jt*_ the fixed environment-by-group interaction term, *G*_*ji*_ the random environment-specific genetic effect of the *i*th genotype in the *j*th environment, and ε_*ij*_∼*N*(0,1) are the standard normally distributed and independent random residual error effects (for all *i* = 1,..,*p* and *j* = 1, 2). In contrast to the residual errors, the random genetic effects are not necessarily independent for all *i* and *j*. The bivariate random genetic effect *G*_*i*_=(*G*_1*i*_,*G*_2*i*_)*T* of the *i*th genotype follows a bivariate normal distribution *G*_*i*_∼*N*(0, ∑*_E_*) where the unstructured variance–covariance matrix ∑*_E_* allows for heterogeneity of genetic variances in both environments.

Fitting the described mixed model using the “asreml” Rpackage (Butler, 2020) leads to estimations for the mean of each genotype group (BLUEs for PJ, PZ, PJ′, PZ′, H_1_, and both checks) and to predictions for each of the genotypes (BLUPs for H_1_ offspring, parent, and foundation clones), both across environments and for each population and trait. The heterosis increment was estimated by calculating for each trait a new variable on the basis of the estimated mid-offspring (i.e., BLUEs for each individual hybrid family performance) minus the estimated mid-parent value [(i.e., mean of the BLUEs for PJ′ and PZ′ parents] corresponding to each mid-offspring and hybrid family, respectively. This new variable representing the difference between a mid-offspring and a mid-parent performance (heterotic gain in each individual hybrid family) was recorded as percentage for each environment, followed by estimation of the heterosis increment for the hybrid group across environments using BLUPs, which represents the heterotic gain of PJ′ and PZ′ crossings. The total genetic gain was estimated as the difference of the BLUE for the hybrid group and the mean of the BLUEs for the PJ and PZ groups. The confidence limits were approximated using the standard errors for the BLUEs and assuming normality.

To obtain the estimations of the variance components, the following mixed model was used:


$${\hat{\mu }}_{i\,\left(j\right)}=\mu +F_{f}+G\,{\left(F\right)}_{i}+e_{j}+F\,e_{f\,j}+G\,e\,{\left(F\right)}_{i\,j}+\varepsilon_{i\,j}$$

where ${\hat{\mu }}_{i\,\left(j\right)}$ (*j* = 1,2;*i* = 1,..,*p*_*h*_) is the reweighted BLUE of the *i*th hybrid genotype (belonging to the *f*th genetic family; not explicitly denoted) in the *j*th environment, μ the overall mean, $F_{f}\,∼\,N\,\left(0,\sigma_{F}^{2}\right)$ is the random family effect, $G\,{\left(F\right)}_{i}\,∼\,N\,\left(0,\sigma_{G\,\left(F\right)}^{2}\right)$ the random genotype (within family) effect, $e_{j}\,∼\,N\,\left(0,\sigma_{L}^{2}\right)$ the random environment (location) effect, $F\,e_{f\,j}∼\,N\,\left(0,\sigma_{F\,x\,L}^{2}\right)$ the random family-by-location interaction effect, $G\,e\,{\left(F\right)}_{i\,j}\,∼\,N\,\left(0,\sigma_{G\,x\,L\,\left(F\right)}^{2}\right)$ the random genotype-by-location interaction effect, and ε_*ij*_ ∼ *N*(0, 1) are the standard normally distributed and independent random residual error effects (for all *i* = 1,..,*p*_*h*_ and *j* = 1, 2). The same weighting scheme as above, based on the first-stage analysis, was used – Method 2 of Möhring and Piepho (2009).

## Results

### Within-Location Analysis Revealed a Good Quality of Phenotypic Data

Field trials for the three H_1_ populations had low-to-medium location-specific heritabilities for yield traits (0.16–0.70; Table 1). For quality traits, all location-specific heritabilities were medium to high (0.55–0.90). Across most trials, the heritability for harvest index was the largest heritability among the yield traits. The main trends for storage root yield were low performance in the off-season trial at the 90-day harvest (H_1_-O-WAE Cañete: 7.6 t ha^–1^, Peruvian winter season), moderate performance in the on-season trial at the 90-day harvest (H_1_-O-WAE Satipo: 26.3 t ha^–1^), and moderate-to-high performance in the off- and on-season trials at the 120-day harvest (H_1_-O-WAE Cañete: 16.4 t ha^–1^, Satipo: 53.3 t ha^–1^, respectively). The H_1_-O-NSSP and H_1_-O-HIFE trials (on-season with 120-day harvest) were in alignment with the storage root yield trend in H_1_-O-WAE on-season (35.9–51.9 t ha^–1^), except for one trial (H_1_-O-NSSP Satipo: 17.5 t ha^–1^). The foliage yields were high (up to 90.5 t ha^–1^) even for the 90-day harvest (H_1_-O-WAE Satipo: 73. t ha^–1^), except for the off-season (Cañete during Peruvian winter). The dry matter contents were usually medium (24–26.2%), with some trials displaying the elevated estimates (H_1_-O-NSSP Satipo of 27.7%; H_1_-O-WAE Cañete at the 90- and 120-day harvests of 27.4 and 29%, respectively). The β-carotene contents varied across sites with a range of 17.3–37.9-mg 100 g^–1^ dwb. The COSW score in H_1_-O-NSSP was medium (5.71, only estimated at Satipo). The mean iron estimates in H_1_-O-HIFE trials were elevated ≅ (25 mg kg^–1^dwb).

**TABLE 1:** Mean of best linear unbiased estimations ($\bar{x}$ BLUEs) and location-specific heritabilities (h^2^) for observed traits in three OFSP H_1_ population experiments conducted at two locations in Peru (Cañete and Satipo), based on the OFSP H_1_ population (WAE: *N* = 9,881; NSSP: *N* = 3,742; HIFE: *N* = 3,292), parents (WAE: 41 PJ′ and 41 PZ′; NSSP: 25 PJ′ and 28 PZ′; HIFE: 28 PJ′ and 28 PZ′), founder clones (49 PJ and 31 PZ), and checks (Cemsa_74-228 and Dagga); OFSP H_1_ populations: WAE, wide adaptation and earliness; NSSP, non-sweet sweetpotato; HIFE, high iron; RYTHA, storage root yield; NCRPL, number of commercial roots per plant; FYTHA, foliage yield; HI, harvest index; DM, root dry matter; BC, β-carotene; COSW, sweetness taste after cooking; STA, root starch; SUC, root sucrose; FE, root iron.

		Cañete (arid Pacific coast)		Satipo (humid tropics)	
			
Trial (harvest day)	Trait	$\bar{x}$ BLUEs	h^2^	$\bar{x}$ BLUEs	h^2^
WAE (90)	RYTHA (t ha^–1^)	7.6	0.45	26.2	0.32
	NCRPL	1.02	0.40	2.74	0.39
	FYTHA (t ha^–1^)	11.1	0.34	73.0	0.28
	HI (%)	42.2	0.49	27.1	0.51
	DM (%)	27.4	0.72	24.9	0.77
	BC (mg 100 g^–1^)	19.0	0.90	21.6	0.84
WAE (120)	RYTHA (t ha^–1^)	16.4	0.50	53.3	0.18
	NCRPL	2.13	0.35	4.22	0.28
	FYTHA (t ha^–1^)	17.8	0.61	90.5	0.21
	HI (%)	49.5	0.51	39.0	0.47
	DM (%)	29.0	0.76	26.1	0.75
	BC (mg 100 g^–1^)	20.0	0.87	25.7	0.86
NSSP (120)	RYTHA (t ha^–1^)	40.5	0.49	17.4	0.16
	NCRPL	3.31	0.41	2.20	0.37
	FYTHA (t ha^–1^)	45.1	0.64	70.6	0.22
	HI (%)	49.5	0.48	21.0	0.44
	DM (%)	25.1	0.68	27.7	0.76
	BC (mg 100 g^–1^)	17.3	0.55	24.0	0.85
	COSW (scale 1–9)	NA	NA	5.71	0.23
HIFE (120)	RYTHA (t ha^–1^)	35.9	0.70	51.9	0.66
	NCRPL	2.83	0.51	4.10	0.53
	FYTHA (t ha^–1^)	46.1	0.47	35.3	0.64
	HI (%)	44.3	0.59	61.1	0.64
	DM (%)	24.1	0.74	24.0	0.81
	STA (%)	50.8	0.88	49.1	0.93
	SUC (%)	17.0	0.89	17.7	0.92
	BC (mg 100g^–1^)	37.9	0.89	35.1	0.90
	FE (mg kg^–1^)	21.9	0.70	21.3	0.80

### Hybrids Outyielded the Average Performance of Parental Populations

The storage root yield in H_1_-O-WAE hybrids (742 families) at the 90- and 120-day harvests was superior to both parental groups at Satipo (Table 2) but not at Cañete, (Peruvian winter) where storage root yield was close to the mean of parental groups. With respect to H_1_-O-NSSP and H_1_-O-HIFE hybrids (336 and 272 families), there were higher storage root yields compared to both parental groups at both experimental sites. The number of commercial roots per plant exhibited a similar pattern to the storage yield for hybrid superiority compared to parental groups (with remarkable estimates above four in H_1_-O-WAE and H_1_-O-HIFE for the 120-day harvest at Satipo). Harvest index was also often higher in hybrid populations than in both parental groups or close to the higher performing parental group. However, foliage yield in hybrid populations was an exception, with a mean close to the mean of both parental groups. Root quality traits of the hybrid populations exhibited means close to the means of parental groups, except for β-carotene content for which hybrid means were close to or slightly below parental group PJ′. The mid-parent–mid-offspring correlation exceeded zero for all hybrid populations and experimental sites (lowest value close to 0.2). Yield traits exhibited low-to-medium mid-parent–mid-offspring correlations (*r* = 0.186–0.590), whereas, for quality traits, these correlations were medium to high (*r* ≤ 0.736), except for β-carotene contents for H_1_-O-NSSP clones at Cañete with *r* = 0.369. Dry matter content had the highest mid-parent–mid-offspring association among quality traits.

**TABLE 2:** Mean of parental BLUEs (for PJ’ and PZ’ separately), mean of mid-offspring across all families (mid-offspring estimated as mean of family-specific hybrid BLUEs), and mid-parent-mid-offspring correlation at experimental sites for observed traits in three OFSP H_1_ populations evaluated at two locations in Peru (Cañete and Satipo): WAE, wide adaptation and earliness (*N* = 9,881); NSSP, non-sweet sweetpotato (*N* = 3,742); HIFE, high iron (*N* = 3,292); RYTHA, storage root yield; NCRPL, number of commercial roots per plant; FYTHA, foliage yield; HI, harvest index; DM, root dry matter; BC, β-carotene; COSW, sweetness taste after cooking; STA, root starch; SUC, root sucrose; FE, root iron; MP-MO r_*p*_, mid-parent–mid-offspring Pearson correlation.

OFSP H_1_ hybrid population (harvest day)	Trait	Cañete (arid Pacific coast)				Satipo (humid tropics)			
			
		PJ′ $\bar{x}$ Parent BLUEs	PZ′ $\bar{x}$ Parent BLUEs	PJ′ × PZ′ Mid-offspring $\bar{x}$ BLUEs	MP-MO r_*p*_	PJ′ $\bar{x}$ Parent BLUEs	PZ′ $\bar{x}$ Parent BLUEs	PJ′ × PZ′ Mid-offspring $\bar{x}$ BLUEs	MP-MO r_*p*_
WAE (90)	RYTHA (t ha^–1^)	6.4	8.2	7.6	0.299	22.9	15.2	26.3	0.194
	NCRPL	0.95	1.09	1.02	0.377	2.71	1.97	2.75	0.207
	FYTHA (t ha^–1^)	9.7	15.1	11.1	0.422	54.4	85.1	73.0	0.382
	HI (%)	38.8	38.8	42.2	0.414	28.6	16.6	27.3	0.462
	DM (%)	27.7	27.6	27.5	0.638	23.7	25.5	24.9	0.701
	BC (mg 100 g^–1^)	20.4	21.2	18.8	0.667	24.5	21.4	21.4	0.589
WAE (120)	RYTHA (t ha^–1^)	14.0	18.9	16.4	0.346	48.7	41.2	53.6	0.209
	NCRPL	1.99	2.26	2.13	0.346	4.17	3.66	4.24	0.272
	FYTHA (t ha^–1^)	13.6	25.5	17.8	0.501	63.7	113.8	90.6	0.423
	HI (%)	49.8	45.1	49.4	0.508	43.4	28.8	39.2	0.367
	DM (%)	29.1	29.0	29.0	0.736	25.5	26.5	26.2	0.630
	BC (mg 100 g^–1^)	21.1	24.0	20.0	0.660	30.7	27.3	25.6	0.654
NSSP (120)	RYTHA (t ha^–1^)	25.7	32.7	41.5	0.186	10.4	14.2	17.7	0.356
	NCRPL	2.52	2.77	3.35	0.170	1.90	1.63	2.22	0.275
	FYTHA (t ha^–1^)	28.0	44.6	45.3	0.441	49.7	97.2	70.1	0.248
	HI (%)	48.9	43.5	50.1	0.332	20.7	14.5	21.3	0.323
	DM (%)	25.6	23.9	25.1	0.637	27.8	26.1	27.6	0.647
	BC (mg 100 g^–1^)	18.6	21.9	17.4	0.369	24.0	30.7	24.7	0.476
	COSW (scale 1–9)	NA	NA	NA	NA	5.64	5.66	5.72	0.193
HIFE (120)	RYTHA (t ha^–1^)	19.6	28.1	36.4	0.522	36.5	45.1	52.2	0.590
	NCRPL	1.99	2.39	2.85	0.451	3.43	3.72	4.12	0.396
	FYTHA (t ha^–1^)	30.7	49.1	46.5	0.467	25.4	36.3	35.4	0.398
	HI (%)	38.9	35.9	44.7	0.507	60.6	53.7	61.3	0.507
	DM (%)	25.3	23.8	24.0	0.647	24.7	23.8	24.0	0.582
	STA (%)	50.4	51.2	50.5	0.525	47.3	48.9	48.9	0.485
	SUC (%)	18.2	15.8	17.2	0.713	20.1	16.8	17.9	0.637
	BC (mg 100g^–1^)	42.8	46.2	38.4	0.507	38.3	42.3	35.3	0.503
	FE (mg kg^–1^)	23.0	20.4	22.0	0.425	23.2	20.0	21.4	0.445

### Significant Commercial Heterotic Gains in Hybrid Populations

The mean estimates of each individual H_1_ offspring cross combination, compared to their individual mid-parent estimates across sites, exhibited in each hybrid population positive differences for yield traits, i.e., storage root yield, number of commercial roots per plant, foliage yield, and harvest index (Table 3). These differences are significant at the 5% level, except for foliage yield in one trial (H_1_-O-WAE at the 120-day harvest). The average storage root yield advantage in H_1_ offspring compared to its parents was 4.4 (H_1_-O-WAE at 90-day harvest), 5.6 (H_1_-O-WAE at 120-day harvest), 9 (H_1_-O-NSSP), and 10 t ha^–1^ (H_1_-O-HIFE). This corresponds to heterotic gains of 35.2, 19, 43.5, and 29%, respectively, in the H_1_ populations investigated (Table 3). The 95%-confidence limit (CL) of heterosis increment for storage root yield was about ±3%. The magnitude of heterosis for commercial root yield was larger compared to the number of commercial roots per plant and harvest index, whereas foliage yield had the smallest heterosis increments. For all quality traits, the difference between H_1_ offspring and their mid-parent estimates across sites in each hybrid population was usually small and not significant (at the 5% level). The 95% CLs for heterosis, thus, overlapped with zero. However, β-carotene content was an exception with that for hybrid offspring, amounting to −10% compared to mid-parent estimates. Note that the PJ′ female parent β-carotene estimates for all generated hybrid populations (H_1_-O-WAE at the 90- and 120-day harvests, H_1_-O-NSSP, and H_1_-O-HIFE) were lower (20.6, 23.9, 20.3, and 39.3 mg 100 g^–1^, respectively) compared to the PZ′ male parent estimates (22.1, 26.3, 25.7, and 43 mg 100 g^–1^, respectively) (results not presented). In all H_1_ populations, the β-carotene offspring estimates were close to the female parent PJ′ and, thus, clearly lower than the mid-parent estimate (about 2–6 mg 100 g^–1^ lower than male parent PZ′).

**TABLE 3:** Estimations of offspring mean (BLUE for the H_1_ mean) and mid-parent (MP) mean ((PJ’ + PZ’)/2 based on BLUEs for the PJ’ and PZ’ group means) with average heterosis increment in H_1_ offspring, *p*-value of *t*-test statistic for heterosis increment = 0, and PJ’ × PZ’ heterosis increment in % across experimental sites for observed traits in three OFSP H_1_ populations: WAE, wide adaptation and earliness (*N* = 9,881); NSSP, non-sweet sweetpotato (*N* = 3,742); HIFE, high iron (*N* = 3,292); RYTHA, storage root yield; NCRPL, number of commercial roots per plant; FYTHA, foliage yield; HI, harvest index; DM, root dry matter; BC, β-carotene; COSW, sweetness taste after cooking; STA, root starch; SUC, root sucrose; FE, root iron.

OFSP H_1_ population (harvest day)	Trait	PJ′ × PZ′ H_1_ offspring (BLUE)	Parental clones (PJ′ + PZ′)/2 (BLUEs)	Average H_1_ offspring heterosis increment	Heterosis increment = 0 *p*-value | *t*|	Average H_1_ heterosis increment in %	95% CI for average H_1_ heterosis increment in %
WAE (90)	RYTHA (t ha^–1^)	17.0	13.2	3.76	<0.001	28.5	[15.8, 41.1]
	NCRPL	1.89	1.69	0.200	0.023	11.8	[1.6, 22.0]
	FYTHA (t ha^–1^)	42.1	41.1	1.03	0.575	2.5	[−6.3, 11.3]
	HI (%)	34.8	30.6	4.14	0.003	13.5	[4.5, 22.5]
	DM (%)	26.2	26.2	0.01	0.973	0.0	[−2.8, 2.9]
	BC (mg 100 g^–1^)	20.1	21.7	−1.50	0.381	−6.9	[−22.5, 8.6]
WAE (120)	RYTHA (t ha^–1^)	35.0	30.8	4.17	0.004	13.5	[4.2, 22.8]
	NCRPL	3.18	3.03	0.153	0.132	5.0	[−1.5, 11.6]
	FYTHA (t ha^–1^)	54.2	54.2	0.02	0.994	0.0	[−10.0, 10.0]
	HI (%)	44.4	41.8	2.56	0.076	6.1	[−0.6, 12.9]
	DM (%)	27.6	27.6	−0.03	0.930	−0.1	[−2.9, 2.7]
	BC (mg 100 g^–1^)	22.7	25.7	−3.00	0.084	−11.7	[−24.9, 1.6]
NSSP (120)	RYTHA (t ha^–1^)	29.4	20.8	8.58	<0.001	41.2	[27.3, 55.1]
	NCRPL	2.78	2.22	0.56	<0.001	25.3	[13.4, 37.1]
	FYTHA (t ha^–1^)	58.1	55.0	3.07	0.410	5.6	[−7.7, 18.8]
	HI (%)	35.6	32.0	3.61	0.015	11.3	[2.2, 20.4]
	DM (%)	26.4	25.8	0.59	0.237	2.3	[−1.5, 6.1]
	BC (mg 100 g^–1^)	20.5	23.8	−3.32	0.065	−13.9	[−28.7, 0.9]
	COSW (scale 1–9)	5.72	5.67	0.049	0.753	0.9	[−4.5, 6.2]
HIFE (120)	RYTHA (t ha^–1^)	44.6	32.3	12.27	<0.001	38.0	[23.6, 52.3]
	NCRPL	3.50	2.89	0.606	<0.001	21.0	[11.2, 30.7]
	FYTHA (t ha^–1^)	40.9	35.4	5.52	0.029	15.6	[1.6, 29.6]
	HI (%)	53.2	47.5	5.70	<0.001	12.0	[5.2, 18.8]
	DM (%)	24.0	24.4	−0.40	0.393	−1.7	[−5.5, 2.1]
	STA (%)	49.7	49.4	0.33	0.782	0.7	[−4.1, 5.5]
	SUC (%)	17.5	17.7	−0.29	0.742	−1.6	[−11.4, 8.1]
	BC (mg 100 g^–1^)	36.6	42.5	−5.87	0.012	−13.8	[−24.6, −3.0]
	FE (mg kg^–1^)	21.7	21.7	0.05	0.919	0.2	[−4.0, 4.4]

### Genetic Variation Is Larger Within Than Among H_1_ Families

For all traits, estimated $\sigma_{F}^{2}$ and $\sigma_{G\,\left(F\right)}^{2}$ in each H_1_ population were significant (CL_*lb*_ larger than zero) (Table 4), and $\sigma_{G\,\left(F\right)}^{2}$ was considerably larger than $\sigma_{F}^{2}$. For storage root yield $\sigma_{G\,\left(F\right)}^{2}$, was usually three to five times larger than $\sigma_{F}^{2}$ with values up to 9.3 times (H_1_-O-NSSP). In all H_1_ populations, yield traits exhibited very large ranges of $\sigma_{G\,\left(F\right)}^{2}$ (e.g., storage root yield and harvest index, 15.3–169.3 t^2^ ha^–2^ and 86.0–109.8%^2^, respectively). For quality traits $\sigma_{G\,\left(F\right)}^{2}$, was considerably larger than $\sigma_{F}^{2}$ (e.g., key quality traits dry matter and β-carotene ratios of $\sigma_{G\,\left(F\right)}^{2}$ and $\sigma_{F}^{2}$ were in the ranges of 2.5–3.2 and 3.4–6.9, respectively). The quality traits exhibited large $\sigma_{G\,\left(F\right)}^{2}$ for the key traits, dry matter contents of 7.1–9.4%^2^ and β-carotene contents of 113.2–252.1 mg^2^ 100 g^–2^), except for COSW and iron (0.16 COSW score^2^ in H_1_-O-NSSP, and 9.4 mg^2^ kg^–2^ iron in H_1_-O-HIFE). The $\sigma_{L}^{2}$ of yield traits was usually much larger than $\sigma_{F}^{2}$ and $\sigma_{G\,\left(F\right)}^{2}$ whereas, for quality traits, it was considerably smaller than $\sigma_{G\,\left(F\right)}^{2}$ and, most often, smaller than $\sigma_{F}^{2}$. The genotype-by-environment interactions $\sigma_{F\,x\,L}^{2}$ and $\sigma_{G\,\left(F\right)\,x\,L}^{2}$ were small to medium for yield traits [relative to $\sigma_{F}^{2}$ and $\sigma_{G\,\left(F\right)}^{2}$ with $\sigma_{F\,x\,L}^{2}$ ratios of nearly zero to 2.2 and $\sigma_{G\,\left(F\right)\,x\,L}^{2}$ ratios usually < 0.5], except for the number of commercial roots per plant with ratios close to 1. All quality traits had $\sigma_{F\,x\,L}^{2}$ and $\sigma_{G\,\left(F\right)\,x\,L}^{2}$ smaller than $\sigma_{F}^{2}$ and $\sigma_{G\,\left(F\right)}^{2}$ respectively.

**TABLE 4:** Variance component estimates with 95% CL limits in square parentheses for observed traits in three OFSP H_1_ populations for observed traits; OFSP H_1_ populations: WAE, wide adaptation and earliness (*N* = 9,881); NSSP, non-sweet sweetpotato (*N* = 3,742); and HIFE, high iron (*N* = 3,292); $\sigma_{F}^{2}$, $\sigma_{G\,\left(F\right)}^{2}$, $\sigma_{L}^{2}$, $\sigma_{F\,x\,L}^{2}$, and $\sigma_{G\,x\,L\,\left(F\right)}^{2}$, variance components due to H_1_ families, genotypes within H_1_ families, locations, interaction of H_1_ families by location, and interaction of genotypes within H_1_ families, respectively; RYTHA, storage root yield; NCRPL, number of commercial roots per plant; FYTHA, foliage yield, BC, HI, harvest index; DM, root dry matter; BC, β-carotene; COSW, sweetness taste after cooking; STA, root starch; SUC, root sucrose; FE, root iron; NA, not available.

OFSP H_1_ population (harvest day)	Trait	$\sigma_{F}^{2}$	$\sigma_{G\,\left(F\right)}^{2}$	$\sigma_{L}^{2}$	$\sigma_{F\,x\,L}^{2}$	$\sigma_{G\,x\,L\,\left(F\right)}^{2}$
WAE (90)	RYTHA (t^2^ha^–2^)	3.1 [2.1, 4.0]	15.3 [14.3, 16.4]	174.9 [0, 656.0]	2.1 [1.1, 3.0]	<0.001 NA
	NCRPL	0.04 [0.03, 0.06]	0.22 [0.18, 0.26]	1.50 [0, 5.6]	0.03 [0.01, 0.05]	0.23 [0.19, 0.27]
	FYTHA (t^2^ha^–2^)	10.1 [8.4, 11.8]	28.5 [25.9, 31.1]	1928.7 [0, 7275.0]	<0.001 NA	<0.001 NA
	HI (%^2^)	28.8 [23.5, 34.0]	86.0 [79.1, 92.9]	111.9 [0, 420.4]	9.7 [6.8, 12.6]	30.3 [23.7, 37.0]
	DM (%^2^)	2.8 [2.4,3.2]	7.1 [6.8, 7.5]	3.5 [0, 13.3]	0.4 [0.3, 0.6]	1.4 [1.1, 1.6]
	BC (mg^2^ 100 g^–2^)	44.5 [37.8, 51.3]	152.3 [145.1, 159.4]	2.7 [0, 10.1]	4.4 [2.7, 6.1]	57.6 [53.7, 61.5]
WAE (120)	RYTHA (t^2^ha^–2^)	16.1 [12.2, 20.0]	61.2 [57.3, 65.2]	691.2 [0, 2607.4]	0.3 [0, 3.8]	<0.001 NA
	NCRPL	0.04 [0.01, 0.06]	0.33 [0.27, 0.40]	2.20 [0, 8.28]	0.08 [0.05, 0.11]	0.29 [0.22, 0.37]
	FYTHA (t^2^ha^–2^)	38.2 [32.4, 44.1]	143.9 [136.6, 151.2]	2652.6 [0, 10005.7]	<0.001 NA	<0.001 NA
	HI (%^2^)	33.1 [27.3, 38.9]	102.5 [95.0, 109.9]	53.7 [0, 202.8]	9.7 [6.8, 12.6]	22.9 [16.1, 29.8]
	DM (%^2^)	3.3 [2.8, 3.7]	8.2 [7.8, 8.5]	4.1 [0, 15.4]	0.4 [0.3, 0.5]	1.0 [0.9, 1.2]
	BC (mg^2^ 100 g^–2^)	45.6 [38.8, 52.3]	158.4 [151.3, 165.4]	14.4 [0, 54.4]	2.6 [1.2, 4.0]	59.0 [55.3, 62.7]
NSSP (120)	RYTHA (t^2^ha^–2^)	9.9 [2.0, 17.7]	91.3 [79.1, 103.5]	279.1 [0, 1050.7]	18.8 [11.0, 26.7]	<0.001 NA
	NCRPL	0.05 [0, 0.10]	0.45 [0.34, 0.55]	0.65 [0, 2.45]	0.11 [0.06, 0.17]	0.50 [0.38, 0.63]
	FYTHA (t^2^ha^–2^)	74.2 [45.6, 102.9]	434.1 [400.8, 467.3]	314.6 [0, 1188.1]	35.5 [13.0, 58.0]	<0.001 NA
	HI (%^2^)	19.0 [12.5, 25.4]	81.9 [73.9, 89.9]	406.9 [0, 1533.4]	10.0 [6.1, 13.9]	<0.001 NA
	DM (%^2^)	2.9 [2.2, 3.6]	9.2 [8.6, 9.9]	3.6 [0, 13.5]	0.2 [0.1, 0.4]	0.6 [0.2, 0.9]
	BC (mg^2^ 100 g^–2^)	16.3 [9.9, 22.7]	113.2 [101.6, 124.8]	22.0 [0, 83.1]	5.8 [1.9, 9.7]	64.8 [55.9, 73.7]
	COSW (scale^2^ 1–9)	0.10 [0.02, 0.18]	0.16 [0.01, 0.30]	NA	NA	NA
HIFE (120)	RYTHA (t^2^ha^–2^)	57.1 [40.9, 73.4]	169.3 [149.9, 165.8]	125.4 [0, 473.3]	13.9 [6.6, 21.2]	148.3 [130.8, 165.8]
	NCRPL	0.16 [0.10, 0.23]	0.58 [0.48, 0.67]	0.80 [0, 3.03]	0.08 [0.03, 0.12]	0.61 [0.51, 0.72]
	FYTHA (t^2^ha^–2^)	36.8 [21.7, 51.9]	249.0 [225.7, 272.2]	59.2 [0, 224.0]	23.2 [13.7, 32.6]	46.1 [27.7, 64.6]
	HI (%^2^)	22.6 [15.8, 29.4]	109.8 [101.6, 118.0]	136.9 [0, 516.1]	5.0 [2.5, 7.5]	<0.001 NA
	DM (%^2^)	2.9 [2.3, 3.6]	9.4 [8.9, 10.0]	<0.001 [0, 0.01]	0.11 [0.03, 0.19]	0.02 [0, 0.22]
	STA (%^2^)	13.7 [10.2, 17.2]	67.2 [63.4, 71.0]	1.6 [0, 5.9]	0.3 [0.1, 0.6]	4.3 [3.6, 5.0]
	SUC (%^2^)	9.8 [7.6, 12.0]	31.9 [30.1, 33.8]	0.3 [0, 1.0]	0.3 [0.2, 0.5]	3.3 [2.9, 3.7]
	BC (mg^2^ 100 g^–2^)	42.5 [31.1, 53.9]	252.1 [237.9, 266.3]	3.7 [0, 14.0]	1.2 [0.3, 2.2]	8.5 [5.9, 11.0]
	FE (mg^2^kg^–2^)	1.58 [1.09, 2.05]	9.38 [8.70, 10.06]	0.19 [0, 0.71]	0.25 [0.10, 0.39]	1.77 [1.42, 2.12]
						

### H_1_ and Founder Clone Comparisons Revealed Large Genetic Gains

**FIGURE 2:**
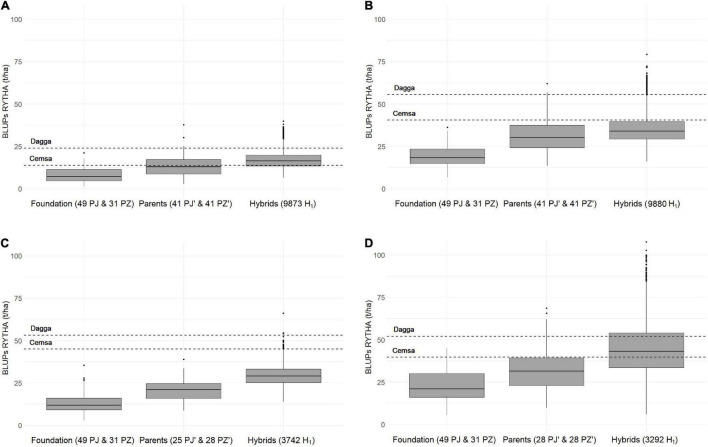
Storage root yield best linear unbiased predictions (BLUPs) for founder clones (foundation), PJ′ and PZ′ hybrid parents (parents), and offspring hybrid clones (hybrids) in three OFSP H_1_ populations [wide adaptation and earliness (WAE) harvested after **(A)** 90 days and **(B)** 120 days, **(C)** non-sweet after cooking (NSSP) harvested after 120 days, and **(D)** high iron (HIFE) harvested after 120 days] together with two check clones (Dagga and Cemsa-74-228) evaluated across two locations in Peru (Cañete on the arid Pacific coast and Satipo in the humid tropics of the Amazon basin).

In all H_1_ populations (PJ′ × PZ′), the yield traits exhibited considerably higher offspring means compared to founder clone means (PJ and PZ) (Table 5), except the foliage yield (close or slightly larger than founder clones), and, in all H_1_ populations, offspring clones had greater storage root yields than the best check clone (Figure 2), especially in H_1_-O-WAE at the 90- and 120-day harvests and H_1_-O-HIFE. With respect to the quality traits, the PJ′ × PZ′ hybrid offspring means in H_1_ populations were close to parental mean estimates (PJ′ and PZ′), and slightly lower than founder clones, except for root sucrose, β-carotene, and iron in H_1_-O-HIFE. The response to selection in yield traits after one recurrent selection cycle (comprising selection of intra-gene pool parents on the basis of H_0_ offspring information and *per se* selection within intra-gene pools) was consistently very large (i.e., difference between H_1_ population means and foundation means) for storage root yield (81.5–132.4%), number of commercial storage roots (45.0–82.7%), and harvest index (35.5–53.0%). Foliage yield was an exception with low-to-medium response to selection (6.1–17.6%). The responses to selection in yield traits were significant (a lower-bound CL limit larger than zero), except for foliage yield in H_1_-O-HIFE. There was a considerable confidence in these high gain figures due to the estimates of the lower-bound CL for selection responses, e.g., storage root yield lower-bound CL was 96.4, 66.6, 113.1, and 79.4% for H_1_-O-WAE 90-day harvest, H_1_-O-WAE 120-day harvest, H_1_-O-NSSP, and H_1_-O-HIFE, respectively. For the key quality traits of dry matter and β-carotene contents, the response to selection in H_1_ populations was usually slightly negative (dry matter, −0.6 to −2%; β-carotene, −4.5 to −10.4%), with an absolute decline of −0.2 to −0.5% dry matter and −1.0 to −2.6-mg 100 g^–1^ β-carotene in H_1_ population means. The exception was the H_1_-O-HIFE population, with a pronounced negative response in dry matter content (−13.3%) and a pronounced positive response for β-carotene (22.3%) and iron content (19.4%). Remarkably, the β-carotene and iron H_1_-O-HIFE population means were considerably larger than the means of check clones, i.e., 32.6 mg 100 g^–1^ dwb more β-carotene and 8.2 mg kg^–1^dwb more iron, so that nearly the entire H_1_-O-HIFE population surpassed the mean of the check clones. The highest iron estimate observed was 45.9 mg kg^–1^ dwb iron associated with 31.6 mg kg^–1^ dwb zinc (clone PH17.9239, cross combination PJ14.13983 × PZ14.14454, results not presented). However, concerning dry matter, only a few H_1_-O-HIFE clones surpassed the check mean (3%). The response to selection for COSW (0.7%) in H_1_-O-NSSP was close to zero and not significant, but nearly 80% of H_1_-O-NSSP offspring clones exhibited desirable lower COSW scores compared to the check clone mean.

**TABLE 5:** Foundation mean performance (based on BLUEs for the PJ and PZ group means), parental mean (based on BLUEs for the PJ′ and PZ′ group means), offspring mean (BLUE for the H_1_ mean), mean check clones (based on BLUEs for Dagga and Cemsa), genetic gain due to heterosis increment, total genetic gain in H_1_ offspring population relative to 49 PJ and 31 PZ foundation clones, and a 95% confidence interval for the total genetic gain, and the calculated frequency of offspring clones that are predicted to be superior to the estimated mean of the checks, in three OFSP H_1_ populations evaluated at Cañete (arid Pacific coast) and Satipo (humid tropics) in Peru: WAE, wide adaptation and earliness (*N* = 9,881); NSSP, non-sweet sweetpotato (*N* = 3,742); and HIFE, high iron (*N* = 3,292); RYTHA, storage root yield; NCRPL, number of commercial roots per plant; FYTHA, foliage yield; HI, harvest index; DM, root dry matter; BC, β-carotene; COSW, sweetness taste after cooking; STA, root starch; SUC, root sucrose; FE, root iron.

OFSP H_1_ population (harvest day)	Trait	Foundation clones (PJ + PZ)/2 (BLUEs)	Parental clones (PJ′ + PZ′)/2 (BLUEs)	PJ′ × PZ′ H_1_ offspring (BLUE)	Check clones (mean of BLUEs for Dagga and Cemsa)	Genetic gain due to heterosis increment (%)	Total genetic gain after one reciprocal recurrent cycle (%)	95% CI for total genetic gain in %	Frequency of offspring clones superior to checks/varieties to replace (%)
WAE (90)	RYTHA (t ha^–1^)	7.8	13.2	17.0	18.8	28.5	118.8	[96.4,141.2]	31.0
	NCRPL	1.04	1.69	1.89	1.78	11.8	82.7	[65.3,100.0]	55.4
	FYTHA (t ha^–1^)	35.8	41.1	42.1	49.0	2.5	17.6	[7.2,28.0]	20.8
	HI (%)	22.7	30.6	34.8	31.1	13.5	53.0	[40.4,65.6]	64.1
	DM (%)	26.4	26.2	26.2	27.4	0.0	−0.9	[−3.8,2.1]	33.1
	BC (mg 100 g^–1^)	21.7	21.7	20.1	2.3	−6.9	−7.1	[−23.3,9.2]	94.3
WAE (120)	RYTHA (t ha^–1^)	19.2	30.8	35.0	47.9	13.5	82.1	[66.6,97.6]	6.6
	NCRPL	2.20	3.03	3.18	3.07	5.0	45.0	[35.6,54.4]	57.1
	FYTHA (t ha^–1^)	47.1	54.2	54.2	68.2	0.0	15.1	[3.1,27.0]	17.3
	HI (%)	32.8	41.8	44.4	43.9	6.1	35.5	[26.5, 44.5]	55.4
	DM (%)	28.1	27.6	27.6	29.5	−0.1	−2.0	[−4.9, 0.9]	25.4
	BC (mg 100 g^–1^)	25.3	25.7	22.7	2.6	−11.7	−10.4	[−24.3, 3.6]	94.4
NSSP (120)	RYTHA (t ha^–1^)	12.7	20.8	29.4	49.0	41.2	132.4	[113.1, 151.7]	0.3
	NCRPL	1.71	2.22	2.78	2.90	25.3	62.7	[49.7, 75.7]	38.7
	FYTHA (t ha^–1^)	50.6	55.0	58.1	88.1	5.6	14.8	[2.7, 26.9]	7.4
	HI (%)	24.4	32.0	35.6	40.5	11.3	46.0	[36.0, 56.1]	25.1
	DM (%)	26.6	25.8	26.4	27.5	2.3	−0.6	[−3.8,2.5]	34.6
	BC (mg 100 g^–1^)	21.5	23.8	20.5	3.6	−13.9	−4.5	[−19.2, 10.1]	98.1
	COSW (scale 1–9)	5.68	5.67	5.72	5.93	0.9	0.7	[−4.4, 5.8]	80.3^§^
HIFE (120)	RYTHA (t ha^–1^)	22.6	32.3	44.6	45.8	38.0	97.1	[79.4, 114.8]	43.3
	NCRPL	2.22	2.89	3.50	2.63	21.0	57.8	[46.8, 68.8]	87.8
	FYTHA (t ha^–1^)	38.5	35.4	40.9	43.4	15.6	6.1	[−5.0, 17.2]	35.8
	HI (%)	37.2	47.5	53.2	49.1	12.0	42.8	[35.3, 50.3]	68.1
	DM (%)	27.7	24.4	24.0	30.8	−1.7	−13.3	[−16.2, −10.4]	3.0
	STA (%)	57.3	49.4	49.7	65.5	0.7	−13.2	[−16.8, −9.6]	2.8
	SUC (%)	13.9	17.7	17.5	11.6	−1.6	25.8	[15.1,36.6]	19.4^§§^
	BC (mg 100 g^–1^)	30.0	42.5	36.6	4.0	−13.8	22.2	[8.8, 35.7]	92.4
	FE (mg kg^–1^)	18.2	21.7	21.7	13.7	0.2	19.4	[15.0, 23.7]	99.8

*^§^Frequency of hybrids with lower sweetness taste than checks after cooking.*

*^§§^Frequency of hybrids with lower sucrose than checks in raw samples.*

## Discussion

### The Aim of Population Hybrid Breeding in Clonal Crops

Hybrid breeding has led to enormous genetic gains in cereal crops (Duvick, 2005; Yuan, 2017). Hybrid breeding *via* homozygous inbreds is not feasible in clonal autopolyploid crops (Gallais, 2003). Therefore, potato breeders in temperate regions have started to develop commercial hybrid seed varieties at the diploid level (Lindhout et al., 2011; Jansky et al., 2016). Nonetheless, a population hybrid breeding approach can be applied in clonal crops regardless of ploidy level (David et al., 2018). We used three OFSP H_1_ populations, all evaluated at normal harvest (120 days after planting), but the WAE population also evaluated very early (90 days after planting), to estimate the heterosis increments in H_1_ and the responses to selection after a complete RRS cycle (see Figure 1, left side). Genetic gains for yield traits, achieved by population hybrid breeding, were high and exceeded our expectations (Table 5).

This approach is also occasionally used in diploid maize with the aim of breeding the population hybrid varieties (Carena, 2005; Hallauer et al., 2010). However, in clonal crops, this approach “only” aims at improved breeding populations for further clonal selection, so, the final product cannot be called a population hybrid variety (for an overview on hybrid varieties, see Wricke and Weber, 1986). The final product is still a clone variety but was bred on the basis of hybrid breeding principles. We propose the term *clonal hybrid* for such a product. However, elite hybrid populations can be developed in isolation as reproducible intermediate products; such isolations comprise two compatible parents with at least one of these a self-incompatible parent. Improved elite populations are a source of further genetic gain (see Figure 1, right side, not examined in this study). True botanical seed production from elite crosses at scale in isolation allows the following: (i) exploitation of the large segregation variance within hybrid families (Table 4) in local target environments; (ii) rapid dissemination of genetic gains from the population improvement in a breeding network or among partners; and (iii) elimination of time-consuming and expensive virus-cleaning procedures in clones, which can take several years. We think this comprehensive concept around the population hybrid breeding is particularly attractive for publicly funded breeding, aiming at developing countries and operating across regions and sub-regions where only one or very few breeding platforms exist, and countries can utilize the true seeds from such platforms for variety development in their context.

### Experimental Sites to Evaluate Hybrid Populations

The H_1_ populations were evaluated at two contrasting sites in Peru: Cañete on the arid Pacific coast, with similarities to a Mediterranean climate; and Satipo in the humid tropics of the Amazon basin. The CIP has made good experience in evaluating breeding material in these contrasting climates for more than a decade [for more details, see Diaz et al. (2021)]. The trials for each hybid population were conducted at each site in separate but adjacent fields during the same season, except for H_1_-O-WAE early and late harvest in Cañete; due to delays in the availability of field area, this trial partially overlapped with the winter season at the Peruvian coast (night and day temperature of about 11 and 19°C, respectively). This explains the pronounced yield differences between Cañete and Satipo in H_1_-O-WAE for the 90- and 120-day harvests (Tables 1, 2), but also highlights the low temperature adaptation and the earliness of the H_1_ material. The number of offspring clones per cross combination ranged within 8–16, planted without plot replication in 1-m row plots, but parents and founder clones among H_1_ offspring were replicated (eight 1-m row plots), and the two check clones were planted in a grid (comprising about 9% of the experimental area). This resulted in a nearly balanced field area for offspring–parent and offspring–founder clone comparisons and highly precise check clone estimates. Early and late harvest plots in H_1_-O-WAE trials were completely randomized as double plots, which are facilitating the harvest at different time points and the statistical analysis as a repeated measurement design, including correlations between early and normal harvests. Analysis within experimental sites revealed a good quality of phenotypic data (Table 1), with means and heritabilities consistent with previous reports [for an overview, see Grüneberg et al. (2015) as well as Diaz et al. (2021)].

### Genetic Attributes of H_1_ Populations

As expected, the H_1_ populations generally had a higher storage root yield at each site compared to both parental groups (Table 2), but to obtain estimates for heterotic gains, each offspring must be compared with its individual parents (Table 3). The exception was H_1_-O-WAE in the winter season at Cañete with a storage root yield close to parental groups (Table 2) indicates that the material had reached its lower temperature limits (see above in Experimental sites to evaluate hybrid populations). In terms of product development, the most striking attribute was the suitability for 90-day harvest in H_1_-O-WAE (storage root yield, 26.3 t ha^–1^ at Satipo), which represents a fundamental change in a crop that is normally considered early harvested at 120 days in warm environments (Mwanga et al., 2017). The segregation variance in sweetpotato was very large, e.g., storage root yield in H_1_-O-WAE at 90-day harvest with $\sigma_{F}^{2}$ = 3.1 t^2^ ha^–2^ and $\sigma_{G\,\left(F\right)}^{2}$ = 15.3 t^2^ ha^–2^ (Table 4). The H_1_-O-WAE is a new source for selecting varieties for very short crop durations (80–90 days), which are expected to have a large potential impact for use in Asian rice and wheat cropping systems, but also provides new options for sweetpotato in temperate and semiarid climates characterized by short growing seasons. All hybrid populations surpassed the β-carotene biofortification targets at a population level. The desired β-carotene and dry matter content trait associations were easily achieved in all H_1_ populations due to large $\sigma_{F}^{2}$ and $\sigma_{G\,\left(F\right)}^{2}$ of both traits (Table 4). Iron content in H_1_-O-HIFE requires further population improvement because the biofortification target is 60 mg kg^–1^, assuming the 5% iron bioavailability (personal comm. Wolfgang Pfeiffer), and this trait exhibited only a moderate genetic variation with $\sigma_{F}^{2}$ = 1.58 mg^2^ kg^–2^ and $\sigma_{G\,\left(F\right)}^{2}$ = 9.38 mg^2^ kg^–2^. Mid-parent–mid-offspring correlations were medium to high for quality traits (up to *r* = 0.736, Table 2), but low to medium for yield traits, e.g., storage root yield ranged from close to 0.2 up to 0.5. In general, the mid-parent–mid-offspring associations appear to be much lower in hexaploid sweetpotato than in diploid crops, especially for yield traits [for a more detailed discussion, see Grüneberg et al. (2015)].

The heterosis phenomenon significantly contributed to the performance in all three H_1_ populations. Heterosis estimates were obtained by comparing each individual offspring across sites; for our study, the mean of a segregating offspring, with its individual parents, more specifically, the mid-parent value [(PJ′ + PZ′)/2] across sites (Table 3). The null hypothesis of zero difference between offspring and mid-parent means was tested using a paired *t*-test. All yield traits in all H_1_ populations exhibited positive significant differences and, thus, heterosis increments, except for foliage yield in H_1_-O-WAE at the 120-day harvest (Table 3). The heterosis increment in storage root yield for the PJ′ × PZ′ cross was large, with about 30% across the H_1_ populations; range of 19.–43.5% and about ±3% confidence (95% CL). As expected, the storage root yield heterosis estimated in this study was larger compared with H_0_ figures (21.8 and ±4% confidence) reported by Diaz et al. (2021). Also, as expected, the storage root yield heterosis was generally largest among the yield traits. Among quality traits, the difference between mid-parent and mid-offspring was very small and often not significant, with the exception of β-carotene. The H_1_ mean β-carotene contents were significantly lower than mid-parent estimates. In this study, PJ′ was always used as a female parent and usually had lower β-carotene contents than the PZ′ parent – by 2–6 mg 100 g^–1^ across the three H_1_ populations studied. The female parent seemed to have an influence on β-carotene contents of the offspring, as pointed out by Diaz et al. (2021). However, both sets of parents had very high β-carotene contents, and, in this case, this maternal effect seemed of minor importance.

### Genetic Gains for a Complete Reciprocal Recurrent Selection Cycle and Variety Ability of H_1_ Populations

A complete RRS cycle includes (i) selection for intra-gene pool recombination on the basis of hybrid offspring performance (in our case, PJ and PZ founder clones and an H_0_ offspring) and (ii) selection of new parents within intra-gene pools to develop a new hybrid offspring (in our case, PJ′ and PZ′ clones and an H_1_ offspring). Hence, to estimate the genetic gains for one RRS cycle, we had to determine the difference between H_1_ offspring and founder clones. The H_0_ performance reported by Diaz et al. (2021) was considered as a stage gate for embarking toward H_1_ for different product profiles in 2012/2013. The responses to selection in storage root yield in the three H_1_ populations after one RRS cycle surpassed all our expectations (Figure 2). Moreover, hybrid offspring clones surpassed the two outstanding check clones (Dagga and Cemsa_74-228), especially in H_1_-O-WAE at the 90-day harvest and in H_1_-O-HIFE (Figure 2), indicating that our three H_1_ populations exhibited variety ability – a term coined by Gallais (2003). For all yield traits, the response to selection was impressive in all hybrid populations, with response in storage root yields in the range of 81.5–132.4% (Table 5). An exception was foliage yield with low-to-medium responses. However, it is remarkable that no negative response for foliage yield was observed, because there is, generally, a negative genetic correlation between root and foliage yield in sweetpotato – for an overview, see Grüneberg et al. (2015). This achievement is certainly linked to the multi-trait selection procedure used for selection within intra-gene pools (Supplementary Information). The confidence in the genetic gains is high, as shown by the 95% CL estimates for the response to selection (e.g., storage root yield about ±10% to ±15%). The gains observed by RRS were much higher compared to traditional breeding approaches on the basis of recurrent selection with one gene pool, with annual estimates of 0.8–2.5% for storage root yield across different breeding platforms (CIP International Potato Center, 2020). Furthermore, it is remarkable that selecting only a few founder clones for intra-pool recombination to develop H_1_-O-NSSP and H_1_-O-HIFE (five PJ and five PZ clones) had no negative impact on yield traits, which can be explained by the very large effective population size in autopolyploids (Gallais, 2003).

The quality trait performance is crucial for sweetpotato as such traits play a major role in the adoption and desirability of novel varieties by small holder farmers, and, for these traits, we observed both improvements and declines in the hybrid populations that were studied (Table 5). In general, a small negative response for dry matter content was observed (0.2–0.5% dry matter decrease) and a, somewhat, more pronounced negative response for β-carotene contents (usually not below −2.6 mg 100 g^–1^ fwb). Although we consider these decreases in the population means tolerable, in further breeding cycles, declines in root dry matter should be avoided. However, the H_1_-O-HIFE population is an exception with pronounced dry matter content decreases and β-carotene content increases compared to founder clones (−3.7% dry matter and 3.6 mg 100 g^–1^ fwb β-carotene). This could be due to the (i) k_*s*_ setting for the modified Pesek Baker Index in intra-gene pool selections (Supplementary Information 2), (ii) positive genetic association between β-carotene and iron content in OFSP (Grüneberg et al., 2015), and (iii) very high k_*s*_ weight for iron improvement with the intention of demonstrating the possibility of iron biofortification in sweetpotato. The observed response to selection in iron was moderate (19.4%), but, remarkably, almost all H_1_-O-HIFE offsprings exceeded the mean of the checks (99.8% with an H_1_-O-HIFE offspring mean of 8.2-mg kg^–1^ iron above the mean of checks). This is a significant improvement for this micronutrient, which is seriously deficient in food supply (Jongstra et al., 2020). Outstanding clones such as PH17.9239 (45.9 mg kg^–1^ dwb iron and 31.6 mg kg^–1^ dwb zinc, results not presented) are an indication that double or triple biofortification might be possible with further RRS cycles. However, the genetic gain for iron was accompanied by decreases in root starch and increases in root sucrose content, which are both undesirable in regions where sweetpotato is used as a staple food. Response to selection for COSW was close to zero, but we hypothesize a variety ability for this quality trait in H_1_-O-NSSP (80.3% of H_1_-O-NSSP offspring clones exhibited lower COSW scores than the mean of checks). This was despite the very high k_*s*_ weight for less root sucrose in raw storage roots in intra-gene pool selections (Supplementary Information for Modification of the Pesek Baker Index and Selection in intra-gene pools). This indicates that selection for low sugar in raw storage roots had very little effect on sweetness after cooking, and that there is a need to determine individual sugars after cooking or, generally, by sweetness in taste panels. We showed that population hybrid breeding can be very successful with respect to yield traits; however, with respect to quality, multi-trait selection procedures for population improvement should place more emphasis on root dry matter in the next RRS cycles, especially for the product profile of high-iron OFSP.

## Conclusions

There is a considerable amount (20–40%) of heterosis achievable in sweetpotato commercial storage root yield that must be considered when comparing RRS with a recurrent selection using one gene pool. Population hybrid breeding responses to selection in storage root yield, with an RRS cycle of 5–6 years, are equivalent to about 30–50 years of polycross breeding, which is the traditional breeding scheme for sweetpotato. Further research should focus on appropriate multi-trait selection procedures and incorporation of genomic selection in the proposed hybrid breeding scheme. The population hybrid breeding approach is probably also applicable to other clonal crops, such as potato, cassava, banana, yam, and sugar cane.

## Data Availability Statement

The datasets presented in this study can be found in online repositories. The names of the repository/repositories and accession number(s) can be found below: https://sweetpotatobase.org/folder/4366.

## Author Contributions

WG, BD, and JR contributed to the writing of this article. WG, FD, and RE provided design and supervision of field trials. WG, BD, and RE contributed to the data management and statistical analysis. JL, WG, and HC contributed to writing the project proposal to fund this study. WG contributed to the editing of the article and assured compliance with author guidelines. All authors contributed to the article and approved the submitted version.

## Conflict of Interest

The authors declare that the research was conducted in the absence of any commercial or financial relationships that could be construed as a potential conflict of interest.

## Publisher’s Note

All claims expressed in this article are solely those of the authors and do not necessarily represent those of their affiliated organizations, or those of the publisher, the editors and the reviewers. Any product that may be evaluated in this article, or claim that may be made by its manufacturer, is not guaranteed or endorsed by the publisher.
