# Supplementary material for: Heterosis and Responses to Selection in Orange-Fleshed Sweetpotato (Ipomoea batatas L.) Improved Using Reciprocal Recurrent Selection
Source: Front Plant Sci. 2022 Apr 26;13:793904. doi: 10.3389/fpls.2022.793904 (PMC9087839; doi:10.3389/fpls.2022.793904)
Supplement: Supplementary file 1 [file Data_Sheet_1.docx]

**SUPPLEMENTARY MATERIAL**

**Supplement Table S1.** Description of PJ and PZ founder clones (comprising entirely breeding lines of Peruvian origin), pedigree, root flesh color (FC), root skin color (SC), root shape (RS), and plant type (PT); PE, Peru; BL, breeding line; C, cream; DO, deep orange; O, orange; W, white; PI, pink; R, red; P, purple; PR, purple red; B, brown; E, elliptic; LE, long elliptic; R, round; RE, round elliptic; E, erect; SE, semi-erect; SP, spreading; ES, extremely spreading (clones selected for intra-pool crossing for the product profiles O-WAE, O-NSSP, and O-HIFE are in bold, $, or ‡, respectively).

| **Cultivar name or breeding code** | **Population** | **CIP code** | **Female parent** | **Male parent** | **FC** | **SC** | **RS** | **PT** |
| --- | --- | --- | --- | --- | --- | --- | --- | --- |
| **PJ05.012** | PJ05 | 189129.1 | YM89.087 | OP | DO | O | E | SE |
| PJ05.018 | PJ05 | 189148.2 | YM89.133 | OP | O | B | E | SE |
| PJ05.039 | PJ05 | 194515.2 | SR93.048 | OP | O | O | E | SE |
| PJ05.043 | PJ05 | 194539.4 | SR93.103 | OP | O | O | E | SE |
| PJ05.052 | PJ05 | 194540.5 | SR93.120 | OP | DO | PI | RE | SE |
| **PJ05.064** | PJ05 | 189151.3 | YM89.150 | OP | O | B | E | SE |
| PJ05.091 | PJ05 | 189148.2 | YM89.133 | OP | DO | R | E | SE |
| PJ05.108 | PJ05 | 189165.3 | YM89.239 | OP | PO | O | RE | SE |
| PJ05.109 | PJ05 | 189168.3 | YM89.252 | OP | O | O | E | SE |
| **PJ05.114** | PJ05 | 190089.6 | SR90.302 | OP | PO | Y | RE | SE |
| **PJ05.120^§^** | PJ05 | 190094.3 | SR90.322 | OP | PO | O | E | SE |
| **PJ05.124** | PJ05 | 190094.9 | SR90.322 | OP | DO | B | E | SE |
| **PJ05.130** | PJ05 | 194513.2 | SR93.028 | OP | DO | PI | RE | SE |
| **PJ05.171^§^** | PJ05 | 194581.4 | SR93.529 | OP | O | PI | E | SE |
| PJ05.172 | PJ05 | 194583.2 | SR93.540 | OP | DO | Y | LE | SE |
| PJ05.180**^§^** | PJ05 | 189157.6 | YM89.166 | OP | PO | Y | E | SE |
| PJ05.202 | PJ05 | 189127.1 | YM89.081 | OP | DO | R | E | SE |
| **PJ05.210** | PJ05 | 194568.1 | SR93.292 | OP | PO | R | LE | SE |
| **PJ05.212** | PJ05 | 189151.8 | YM89.150 | OP | DO | O | E | SE |
| **PJ05.213** | PJ05 | 194575.8 | SR93.453 | OP | DO | O | E | SE |
| PJ05.214 | PJ05 | 194579.1 | SR93.521 | OP | PO | B | E | SE |
| **PJ05.216**^‡^ | PJ05 | 189153.8 | YM89.158 | OP | DO | O | E | SE |
| PJ05.217^‡^ | PJ05 | 189153.1 | YM89.158 | OP | O | O | E | SE |
| **PJ05.219**^‡^ | PJ05 | 189151.3 | YM89.150 | OP | DO | Y | E | SE |
| **PJ05.220** | PJ05 | 189151.5 | YM89.150 | OP | PO | PI | E | SE |
| PJ05.224 | PJ05 | 189206.1 | YM89.170 | OP | PO | PI | E | SE |
| PJ05.227 | PJ05 | 189164.7 | YM89.236 | OP | O | O | RE | SE |

Supplementary Table S1. Continued.

| **Cultivar name or breeding code** | **Population** | **CIP code** | **Female parent** | **Male parent** | **FC** | **SC** | **RS** | **PT** |
| --- | --- | --- | --- | --- | --- | --- | --- | --- |
| **PJ05.233** | PJ05 | 189165.9 | YM89.239 | OP | DO | B | E | SE |
| **PJ05.235** | PJ05 | 189153.7 | YM89.158 | OP | PO | W | E | SE |
| **PJ05.236** | PJ05 | 189153.2 | YM89.158 | OP | DO | PR | E | SE |
| PJ05.238 | PJ05 | 189123.3 | YM89.074 | OP | O | PI | E | SE |
| PJ05.239 | PJ05 | 189125.2 | YM89.078 | OP | DO | O | E | SE |
| PJ05.240 | PJ05 | 189125.9 | YM89.078 | OP | DO | O | E | SE |
| PJ05.243 | PJ05 | 189165.4 | YM89.239 | OP | DO | PI | E | SE |
| PJ05.245 | PJ05 | 194506.8 | SR93.005 | OP | DO | R | E | SE |
| PJ05.247 | PJ05 | 194531.4 | SR93.082 | OP | DO | R | E | SE |
| **PJ05.248^§^** | PJ05 | 194544.2 | SR93.214 | OP | O | O | R | SE |
| PJ05.251 | PJ05 | 194533.4 | SR93.087 | OP | PO | Y | E | SE |
| **PJ05.253** | PJ05 | 194541.6 | SR93.161 | OP | PO | PI | E | SE |
| PJ05.254 | PJ05 | 194557.1 | SR93.271 | OP | O | B | E | SE |
| **PJ05.255^§^** | PJ05 | 189134.1 | YM89.104 | OP | PO | B | E | E |
| **PJ05.257** | PJ05 | 190094.4 | SR90.322 | OP | PO | R | RE | SE |
| PJ05.258 | PJ05 | 194519.3 | SR93.059 | OP | O | O | E | SE |
| PJ05.302 | PJ05 | 194519.5 | SR93.059 | OP | O | C | E | SE |
| **PJ05.303**^‡^ | PJ05 | 189145.2 | YM89.125 | OP | O | PI | E | SE |
| PJ05.304^‡^ | PJ05 | 189153.3 | YM89.158 | OP | DO | O | LE | SE |
| **PJ05.306** | PJ05 | 189152.3 | YM89.152 | OP | O | O | E | SE |
| **PJ05.324** | PJ05 | 194556.6 | SR93.268 | OP | O | O | E | SE |
| PJ05.347 | PJ05 | 194529.6 | SR93.077 | OP | O | PI | E | SE |
| PZ06.011 | PZ06 | 105152.4 | SR01.020 | 420027 | DO | O | RE | SE |
| PZ06.016 | PZ06 | 105154.1 | SR01.021 | 420027 | O | PI | E | SE |
| PZ06.023^‡^ | PZ06 | 105072.3 | SR01.023 | 192035.5 | DO | O | E | SE |
| PZ06.024 | PZ06 | 105157.1 | SR01.023 | 420027 | DO | O | E | SE |
| PZ06.026 | PZ06 | 105066.1 | SR01.024 | 192035.5 | DO | O | E | SE |
| PZ06.027 | PZ06 | 105066.2 | SR01.024 | 192035.5 | DO | O | RE | SE |
| **PZ06.042^§^** | PZ06 | 105083.1 | SR02.164 | 192035.5 | PO | PI | E | SE |
| **PZ06.048** | PZ06 | 105080.1 | SR02.174 | 192035.5 | DO | R | E | SE |
| PZ06.053 | PZ06 | 105080.6 | SR02.174 | 192035.5 | O | PI | E | SE |
| PZ06.062 | PZ06 | 105167.2 | SR02.175 | 420027 | O | O | E | SE |
| PZ06.070 | PZ06 | 105126.3 | SR02.175 | 420014 | DO | O | E | SE |
| **PZ06.072** | PZ06 | 105229.1 | SR02.177 | 440166 | PO | PI | E | SE |
| PZ06.091 | PZ06 | 105373.1 | 440132 | 440167 | DO | O | LE | SE |
| PZ06.099 | PZ06 | 105458.1 | LM02.082 | 440166 | PO | PI | E | SE |
| PZ06.103 | PZ06 | 105460.1 | LM02.082 | 420027 | PO | R | E | SE |
| PZ06.109 | PZ06 | 105103.1 | LM03.076 | 192035.5 | O | PR | E | SE |
| **PZ06.114** | PZ06 | 105100.1 | LM03.088 | 192035.5 | DO | B | E | SE |

Supplementary Table S1. Continued.

| **Cultivar name or breeding code** | **Population** | **CIP code** | **Female parent** | **Male parent** | **FC** | **SC** | **RS** | **PT** |
| --- | --- | --- | --- | --- | --- | --- | --- | --- |
| **PZ06.115** | PZ06 | 105100.2 | LM03.088 | 192035.5 | DO | O | E | SE |
| **PZ06.120** | PZ06 | 105058.2 | SR01.030 | 192035.5 | DO | P | E | SE |
| **PZ06.124** | PZ06 | 105030.1 | SR02.105 | 187003.1 | DO | PI | E | SE |
| **PZ06.196^§^** | PZ06 | 105212.1 | SR01.020 | 440166 | PO | C | E | SE |
| **PZ06.235** | PZ06 | 105269.1 | 440132 | 440166 | PO | W | E | SE |
| **PZ06.304^§^** | PZ06 | 105495.1 | SR01.009 | 192035.5 | O | R | E | SE |
| **PZ06.307** | PZ06 | 105071.1 | SR01.022 | 192035.5 | DO | PI | E | SE |
| **PZ06.348**^‡^ | PZ06 | 105096.1 | LM03.065 | 192035.5 | O | PI | E | SE |
| **PZ06.349^§^**^‡^ | PZ06 | 105097.1 | LM03.083 | 192035.5 | DO | Y | E | SE |
| **PZ06.353^§^**^‡^ | PZ06 | 105511.1 | SR02.030 | 192035.5 | DO | PI | E | SE |
| **PZ06.359**^‡^ | PZ06 | 105008.1 | SR01.014 | 187003.1 | O | B | E | SE |
| PZ06.360 | PZ06 | 105213.1 | SR01.016 | 440166 | O | O | LE | SE |
| PZ06.385 | PZ06 | 105160.1 | SR02.153 | 420027 | DO | O | RE | SE |
| **PZ06.441** | PZ06 | 105175.1 | SR02.142 | 420027 | O | P | RE | SE |

**Application of Pesek Baker Index**

The Pesek Baker Index (Pesek and Baker 1969) is a multi-trait selection procedure in which, as in other linear selection indices, a phenotypic index is constructed by weighted summation $I=\sum b_{i}x_{i}$ with $b_{i}$ as weights of phenotypic traits $x_{i}$ to be identified, and truncation selection is applied on basis of the $n$ largest index scores out of $N$ candidates. As for other linear selection indices, it scores highest for candidates that have a good overall performance of traits. The Pesek Baker Index determines $b_{i}$ by maximizing the correlation between the phenotypic index $I$ and the aggregated genotype $H=\sum a_{i}g_{i}$, with $a_{i}$ as economic weights by using the restriction of the ratio of desired genetic gains $k_{i}$ for each trait. Then the solution for maximizing the correlation between $I$ and $H$ follows:

$b=G^{-1}k$.

Note that with small $\sigma_{g_{i}}^{2}$ in at least one trait, this method makes selection ineffective, because $b_{i}$ increases in proportion to the decrease of $\sigma_{g_{i}}^{2}$, so that for $\sigma_{g_{i}}^{2}=0$ the corresponding $b_{i}$ is infinity. Moreover, the method becomes very inefficient when breeders set unrealistic desired genetic gains for at least one trait, such as $k_{i}$ beyond the range of ±2$\sigma_{g_{i}}$.

**Modification of the Pesek Baker Index**

The vectors of desired gains$k$ are not directly set by the breeder, they are determined through $k=\sigma_{g_{i}} k_{s}$, with $k_{s}$ referring to the desired gains in standardized units of trait $i$ within the range of ±2 to be set by the breeder. This avoids breeders choosing harmful desired genetic gains that cannot be fulfilled by the genetic variance covariance of the population under selection. The overall response to selection, as well as the response to selection for each trait, is straightforward to determine numerically (see examples in Wricke and Weber 1986), which is recommended if breeders set $k_{s}$ beyond the range ±1.5.

**Selection in intra-genepools**

The k_s_ for selecting among PJ′ and PZ′ clones in each intra-genepool were as follow: (i) O-WAE intra-genepool populations, 1.5, 1.5, and 0.5 for RYTHA, agronomic score value of storage roots, and storage root dry matter (DM), respectively; (ii) O-NSSP intra-genepool populations, 0.5, 0.5, 1.5, 1.5, −1.5, 0.5, and 0.5 for RYTHA, agronomic score value of storage roots, DM, root starch (STA), root sucrose (SUC), root β-carotene (BC), and root iron (FE), respectively; and (iii) O-HIFE intra-genepool populations, 0.5, 0.5, 0.2, 0.2, −0.2, 0.2, and 1.5 for RYTHA, agronomic score value of storage roots, DM, STA, SUC, BC, and FE, respectively. The vector *b* of weights for phenotypic observed traits was determined as described above. In each intra-genepool population, index scores were determined. However, selection was performed in two steps. The first one was on the basis of the Pesek Baker Index scores only, selecting more genotypes as finally desired. In each of the six intra-genepool populations 60, 60, 40, 40, 40, and 40 clones were selected as potential parents for the three H_1_ populations. In a second step, potential parents for H_1_ underwent further selection considering vine strength (to facilitate multiplication), SPVD symptoms, hybrid production traits, and feasibility of parents to be recombined for H_1_. The latter is the true seed production, which was used to discard potential parents with less than four true seeds in a full-sib family and less than four half-sib families. Finally, 41 PJ′ and 41 PZ′, 25 PJ′ and 28 PZ′, and 28 PJ′ and 28 PZ′ clones, were obtained to establish the hybrid populations H_1_-O-WAE, H_1_-O-NSSP, and H_1_-O-HIFE, respectively. The selection intensity and selected fraction $\alpha$, respectively, in C_1_ were very high within intra-genepools and $\alpha$ for each intra-genepool is provided in the main text. In contrast, selection intensity among foundation clones in C_0_ to obtain parents for intra-genepool crossings was very moderate with $\alpha$ of approximately 0.4694 in PJ and 0.5484 in PZ for O-WAE, and correspondingly 0.1020 and 0.1613 for O-NSSP, and 0.1020 and 0.1613 for O-HIFE. Note, for intra-genepool crosses for O-NSSP and O-HIFE, only five clones were selected in each intra-genepool with the intention to demonstrate that even with drastic reduction of parents no bottleneck effects are easily generated in hexaploid sweetpotato.
